# Comprehensive review of ST-segment elevation myocardial infarction: Understanding pathophysiology, diagnostic strategies, and current treatment approaches

**DOI:** 10.1097/MD.0000000000035687

**Published:** 2023-10-27

**Authors:** Chukwuka Elendu, Dependable C. Amaechi, Tochi C. Elendu, Eunice K. Omeludike, Chisom E. Alakwe-Ojimba, Babajide Obidigbo, Otite L. Akpovona, Yuliana Paola Oros Sucari, Sehajmeet Kaur Saggi, Kanishk Dang, Chinedu P. Chinedu

**Affiliations:** a University of California, Santa Cruz, CA; b Igbinedion University, Okada, Nigeria; c Imo State University, Owerri, Nigeria; d University of Port Harcourt, Choba, Nigeria; e Chukwuemeka Odumegwu Ojukwu University, Atughobi, Nigeria; f King’s College Hospital NHS Foundation Trust, London, UK; g Universidad peruana Cayetano Heredia, San Martín de Porres, Peru; h Belarusian State Medical University, Minsk, Belarus; i Nicolae Testemițanu State University of Medicine and Pharmacy, Chişinău, Republic of Moldova; j University of Nigeria Teaching Hospital, Enugu, Nigeria.

**Keywords:** American college of cardiology, American heart association, creatine kinase-MB, European society of cardiology, global registry of acute coronary events, left ventricular ejection fraction, ST-segment elevation myocardial infarction

## Abstract

ST-Segment Elevation Myocardial Infarction (STEMI) is a life-threatening medical emergency characterized by complete coronary artery occlusion, leading to myocardial ischemia and subsequent necrosis. Over the years, STEMI has remained a significant cause of morbidity and mortality worldwide, necessitating a comprehensive understanding of its pathophysiology, accurate diagnostic strategies, and effective treatment approaches.

This review article aims to thoroughly analyze the current knowledge surrounding STEMI, emphasizing key aspects crucial for optimizing patient outcomes. Firstly, the pathophysiology of STEMI will be explored, elucidating the sequence of events from coronary artery plaque rupture to thrombus formation and occlusion. This section will also cover the underlying risk factors contributing to STEMI development, including atherosclerosis, hypertension, and diabetes.

Secondly, the diagnostic modalities for STEMI will be critically evaluated. Traditional electrocardiography remains the cornerstone of STEMI diagnosis. Still, advancements in imaging techniques such as cardiac magnetic resonance imaging and coronary angiography have enhanced accuracy and allow for better risk stratification.

Furthermore, the review will delve into the latest treatment approaches for STEMI. Prompt reperfusion therapy through primary percutaneous coronary intervention or thrombolytic therapy is essential in restoring blood flow and salvaging the jeopardized myocardium. The role of adjunctive medical treatment, including antiplatelet agents, beta-blockers, and statins, will also be discussed in post-STEMI management.

## 1. Introduction

The motivation for this comprehensive review of ST-Segment Elevation Myocardial Infarction (STEMI) stems from the critical importance of this cardiovascular emergency in contemporary medicine.^[1]^ STEMI is a significant cause of worldwide morbidity and mortality, necessitating a thorough understanding of its pathophysiology, diagnosis, and management.^[2]^

One of the primary research gaps this review seeks to address is the need for a comprehensive and up-to-date synthesis of the existing literature and guidelines related to STEMI.^[3]^ While individual studies and guidelines exist, there is a need for more total reviews that bring together the latest research findings and evidence-based recommendations.^[4]^ The objectives of this proposed work are as follows:

### 1.1. To provide a comprehensive overview of the epidemiology and risk factors associated with STEMI

This includes a detailed examination of how the incidence and prevalence of STEMI vary across different populations and regions, considering demographic and lifestyle factors.

### 1.2. To elucidate the pathophysiology of STEMI

We aim to explore the intricate mechanisms behind the development of STEMI, focusing on atherosclerotic plaque rupture, thrombus formation, and myocardial ischemia.

### 1.3. To discuss the diagnostic strategies available for the prompt and accurate identification of STEMI

We will delve into the various diagnostic modalities, including electrocardiography (ECG), cardiac biomarkers, and imaging techniques, highlighting their roles and limitations.

### 1.4. To review the current treatment approaches, including reperfusion therapy and medical interventions

This encompasses a detailed analysis of primary percutaneous coronary intervention (PCI), thrombolytic therapy, adjunctive medical therapies, and their impact on patient outcomes.

### 1.5. To summarize the existing guidelines for managing STEMI

We will provide insights into the recommendations by reputable organizations like the American Heart Association (AHA) and the European Society of Cardiology (ESC) to guide healthcare professionals in the optimal care of STEMI patients.

### 1.6. To assess the prognosis of STEMI patients

This includes examining mortality rates, complications, and factors influencing long-term outcomes.

### 1.7. To explore secondary prevention measures and the role of cardiac rehabilitation

We will emphasize the importance of post-STEMI care in reducing the risk of recurrent cardiovascular events and improving patients’ overall quality of life.

## 2. Review

### 2.1. Related works

*This section discusses relevant studies that have contributed to STEMI research, specifically focusing on imaging and predictive modeling advancements.*

#### 2.1.1. Value of 3-dimensional strain parameters in predicting left ventricular remodeling.

In the study conducted by Xu et al (2017),^[5]^ the authors investigated the value of 3-dimensional strain parameters in predicting left ventricular remodeling after STEMI. Their work provides valuable insights into assessing post-STEMI cardiac remodeling, an essential aspect of patient outcomes. By incorporating advanced imaging techniques and strain analysis, Xu et al demonstrated the potential of these parameters as predictors of remodeling. We recognize the significance of their findings in the context of our review, as they shed light on the evolving landscape of post-STEMI assessment.

#### 2.1.2. Deep learning analysis in coronary computed tomographic angiography imaging.

The study by Han et al (2020)^[6]^ delves into the application of deep learning analysis in coronary computed tomography angiography (CCTA) imaging for the assessment of patients with coronary artery stenosis. While our review primarily focuses on the clinical aspects of STEMI management, we acknowledge the growing importance of advanced imaging techniques in diagnosing and assessing coronary artery disease. Han et al work exemplifies integrating cutting-edge technology, such as deep learning, into cardiovascular imaging, highlighting its potential to enhance diagnostic accuracy and patient care.

### 2.2. Integration into our review

Incorporating these studies into our review broadens the scope of our discussion, encompassing not only the clinical aspects of STEMI but also the technological advancements that contribute to improved patient care. While our primary focus remains on the comprehensive understanding of STEMI, we recognize the value of these related works in showcasing the multidisciplinary nature of research in this field.

By acknowledging these studies, we aim to provide a well-rounded perspective on the evolving landscape of STEMI research, where clinical insights and technological innovations intersect to enhance patient outcomes and the quality of care.

## 3. Methodology

### 3.1. Study design

A comprehensive literature review was conducted to achieve the objectives of this study. The study design involved searching and analyzing relevant scientific publications, peer-reviewed journal articles, clinical practice guidelines, and academic books about STEMI. The literature search was performed from reputable databases, including PubMed, MEDLINE, Google Scholar, and Cochrane Library. The search encompassed articles published from inception until the study’s data collection phase in September 2021.

### 3.2. Data collection

The data collection process involved using specific search terms, including “ST-Segment Elevation Myocardial Infarction,” “STEMI pathophysiology,” “STEMI diagnosis,” “STEMI treatment,” “percutaneous coronary intervention,” “thrombolytic therapy,” and “medical therapy for STEMI.” Additionally, relevant MeSH terms were incorporated to enhance the comprehensiveness of the search.

### 3.3. Inclusion and exclusion criteria

Articles were included if they met the following criteria: Published in English; Peer-reviewed; Focused on the pathophysiology, diagnosis, or treatment of STEMI, and; Contained information contributing to the study’s objectives. Studies not meeting these criteria were excluded from the analysis.

### 3.4. Data analysis

The identified articles were evaluated thoroughly, including abstract and full-text screening, by 2 independent reviewers to ensure consistency and accuracy. Relevant data, such as pathophysiological mechanisms of STEMI, diagnostic strategies, treatment modalities, and outcomes, were extracted from the selected articles. Data from the literature were synthesized and organized based on the predetermined subheadings in the study.

## 4. Results

The literature search yielded 387 articles relevant to the study objectives. After applying the inclusion and exclusion criteria, 81 articles were selected for comprehensive analysis. The data extracted from these articles provided a comprehensive overview of the pathophysiology, diagnostic strategies, and treatment approaches for ST-Segment Elevation Myocardial Infarction.

## 5. Discussion

The findings from the literature review were discussed in detail, addressing each objective of the study. Pathophysiological mechanisms leading to STEMI were explored, highlighting the critical role of atherosclerosis and plaque rupture. Diagnostic strategies were evaluated for accuracy and clinical utility, including ECG, cardiac magnetic resonance imaging (MRI), and coronary angiography. The current treatment approaches, including reperfusion and adjunctive medical therapy, were critically analyzed for their efficacy and potential benefits in managing STEMI patients. The comprehensive literature review provided valuable insights into the pathophysiology, diagnostic strategies, and treatment approaches for ST-Segment Elevation Myocardial Infarction. The study highlighted the importance of prompt recognition, accurate diagnosis, and appropriate management in improving patient outcomes. By addressing the objectives of this study, the findings aim to contribute to the existing knowledge on STEMI and serve as a valuable resource for healthcare professionals involved in the care of STEMI patients.

### 5.1. Definition and epidemiology

STEMI is a severe acute coronary syndrome characterized by complete or near-complete coronary artery occlusion, resulting in prolonged myocardial ischemia and subsequent necrosis of the affected heart muscle.^[7]^ STEMI is typically diagnosed based on electrocardiographic changes, specifically ST-segment elevation, in conjunction with clinical symptoms and cardiac biomarker elevations, such as troponin.

STEMI is a significant public health concern worldwide, accounting for a substantial portion of cardiovascular-related morbidity and mortality. The incidence of STEMI varies across different regions and populations and is influenced by various factors, including age, sex, lifestyle, and access to healthcare.^[8]^

According to global estimates, the incidence of STEMI has shown variability across countries, with higher rates reported in high-income regions. In the United States, approximately 965,000 cases of STEMI are reported annually.^[9]^ In Europe, the overall incidence of STEMI varies among countries, with rates ranging from 80 to 370 cases per 100,000 person-years.^[10]^ Similarly, there is considerable heterogeneity in STEMI incidence in Asia, with reported rates ranging from 33 to 138 cases per 100,000 person-years.^[11]^

Age is a significant determinant of STEMI risk, with incidence rates increasing with advancing age. Elderly individuals, particularly those over 65, have a higher prevalence of comorbidities, including atherosclerotic disease, hypertension, and diabetes, contributing to increased STEMI susceptibility.^[12]^

Sex differences also exist in STEMI epidemiology. Men are generally at a higher risk of developing STEMI than premenopausal women. However, the risk for women increases after menopause, narrowing the gender gap.^[13]^

Furthermore, socioeconomic factors, lifestyle choices, and access to healthcare services influence STEMI prevalence and outcomes. Lower socioeconomic status, unhealthy dietary habits, smoking, physical inactivity, and limited access to timely medical care have been associated with an increased risk of STEMI.

In conclusion, ST-Segment Elevation Myocardial Infarction is a severe and life-threatening cardiovascular condition that significantly burdens global health systems. Understanding its epidemiology and risk factors is crucial for implementing effective preventive strategies and improving the management and outcomes of STEMI patients.

### 5.2. Pathophysiology

The pathophysiology of STEMI is characterized by a complex interplay of atherosclerotic plaque formation, plaque rupture, and subsequent thrombus formation, leading to acute coronary artery occlusion and myocardial ischemia.

#### 5.2.1. Atherosclerosis.

The process of STEMI begins with the development of atherosclerosis, a chronic inflammatory condition characterized by the deposition of lipids, cholesterol, and cellular debris within the intimal layer of the coronary arteries. Over time, these deposits, known as atherosclerotic plaques, grow and lead to the narrowing of the arterial lumen, reducing blood flow to the heart muscle.^[14]^

#### 5.2.2. Plaque rupture.

Vulnerable plaques, characterized by a thin fibrous cap covering a lipid-rich core, are prone to rupture under various triggers, such as increased shear stress, inflammation, and the release of pro-thrombotic substances.^[15]^ The rupture exposes the thrombogenic contents of the plaque to the circulating blood, leading to platelet activation and the formation of a thrombus at the site of the lesion.

#### 5.2.3. Thrombus formation.

The thrombus, composed of platelets and fibrin, rapidly propagates within the coronary artery, resulting in partial or complete occlusion of the vessel. This sudden reduction in blood flow to the downstream myocardial territory leads to acute myocardial ischemia.^[16]^

#### 5.2.4. Myocardial ischemia and infarction.

The affected region of the heart muscle undergoes ischemic injury, with a decrease in oxygen and nutrient supply. As a result, the cardiomyocytes become hypoxic, and their ability to generate energy through aerobic metabolism is impaired. Without prompt reperfusion, the ischemic myocardium progresses to irreversible cell death (infarction).^[17]^

#### 5.2.5. Inflammatory response.

The ischemic insult triggers an inflammatory response involving the release of cytokines and chemokines, leading to the recruitment of immune cells, such as neutrophils and macrophages. These cells exacerbate tissue injury and promote additional plaque instability.^[18]^

#### 5.2.6. Reperfusion injury.

The timely restoration of blood flow, such as through reperfusion therapy, is crucial to salvage the ischemic myocardium. However, reperfusion can also trigger further damage, known as reperfusion injury, characterized by oxidative stress, calcium overload, and inflammation.^[19]^

#### 5.2.7. Myocardial remodeling.

Following the acute phase, the surviving myocardium undergoes a remodeling process involving changes in structure, composition, and function. This process can lead to adverse ventricular remodeling, impairing cardiac function and increasing the risk of heart failure and arrhythmias.^[20]^

In conclusion, the pathophysiology of ST-Segment Elevation Myocardial Infarction involves the progressive development of atherosclerosis, vulnerable plaque rupture, and thrombus formation, ultimately leading to acute coronary artery occlusion and myocardial ischemia. Timely recognition and intervention are essential to mitigate the detrimental effects of STEMI and prevent long-term complications.

### 5.3. Diagnostic strategies

STEMI diagnosis involves a combination of clinical evaluation, ECG, and advanced imaging modalities. Early and accurate diagnosis is crucial for prompt initiation of reperfusion therapy and improved patient outcomes.

#### 5.3.1. Clinical evaluation.

The initial step in diagnosing STEMI involves a thorough clinical assessment, which includes a detailed medical history, physical examination, and evaluation of presenting symptoms. Common symptoms of STEMI include chest pain or discomfort, shortness of breath, diaphoresis, and radiation of pain to the left arm, neck, or jaw. The presence and severity of risk factors such as smoking, hypertension, diabetes, and hyperlipidemia should be assessed.^[21]^

#### 5.3.2. ECG.

ECG remains the cornerstone of STEMI diagnosis. It provides real-time information about the heart’s electrical activity and identifies characteristic changes associated with myocardial ischemia. In STEMI, the ECG typically shows ST-segment elevation of at least 1 mm (0.1 mV) in 2 contiguous leads, accompanied by T-wave inversion or development of pathological Q waves in the corresponding leads.^[8]^ Prompt acquisition and interpretation of ECG findings are essential for early recognition and immediate initiation of reperfusion therapy.

#### 5.3.3. Cardiac biomarkers.

Measuring cardiac biomarkers, such as troponin and creatine kinase-MB, is crucial in STEMI diagnosis. Troponin is highly sensitive and specific for myocardial injury and is usually detectable within a few hours of symptom onset. Elevated levels of troponin in the blood, compatible clinical symptoms, and ECG changes confirm the diagnosis of STEMI.^[22]^

#### 5.3.4. Advanced imaging modalities.

1. Echocardiography: Transthoracic echocardiography is valuable in assessing regional wall motion abnormalities, left ventricular function, and the presence of complications, such as mechanical complications or acute mitral regurgitation.^[23]^
2. Cardiac MRI: Cardiac MRI provides high-resolution heart images and allows for accurate assessment of myocardial viability, infarct size, and microvascular obstruction, aiding in risk stratification, and prognosis prediction.^[24]^
3. Coronary angiography: Coronary angiography is considered the gold standard for visualizing coronary artery anatomy and identifying the site and severity of coronary artery occlusion in STEMI patients. It is essential for guiding subsequent reperfusion therapies, such as primary PCI.^[25]^

#### 5.3.5. Risk stratification.

Risk stratification is essential in STEMI patients to identify those at higher risk of adverse outcomes. Clinical risk scores, such as the Global Registry of Acute Coronary Events score, can assist in estimating the short-term and long-term mortality risk, aiding in decision-making regarding treatment strategies and the level of care required.^[26]^

In conclusion, a comprehensive diagnostic approach is essential for timely and accurate ST-Segment Elevation Myocardial Infarction diagnosis. Clinical evaluation, ECG, and cardiac biomarkers play vital roles in the initial assessment, while advanced imaging modalities, such as echocardiography, cardiac MRI, and coronary angiography, provide valuable information for risk stratification and treatment planning.

### 5.4. Current treatment approaches

STEMI management has evolved significantly over the years, focusing on prompt reperfusion therapy and adjunctive medical treatments to reduce myocardial damage and improve clinical outcomes.

#### 5.4.1. Reperfusion therapy.

1. Primary PCI: Primary PCI is the preferred reperfusion strategy for STEMI patients when it can be performed promptly by an experienced team. This intervention involves the mechanical removal of the thrombus and restoration of blood flow by placing a stent in the occluded coronary artery. Primary PCI has demonstrated superior outcomes compared to thrombolytic therapy in terms of reduced mortality rates and lower risk of reinfarction.^[7]^
2. Thrombolytic therapy: In cases where primary PCI is not readily available or significant delays in performing the procedure, thrombolytic therapy may be considered an alternative reperfusion strategy. Thrombolytic agents, such as tissue plasminogen activator or reteplase, can dissolve the coronary artery thrombus and restore blood flow. However, primary PCI is preferred if performed promptly due to its better outcomes.^[27]^

#### 5.4.2. Adjunctive medical therapy.

1. Dual antiplatelet therapy: All STEMI patients undergoing reperfusion therapy receive dual antiplatelet therapy, typically consisting of aspirin and a P2Y12 receptor inhibitor (clopidogrel, ticagrelor, or prasugrel). Dual antiplatelet therapy reduces the risk of recurrent thrombotic events and stent thrombosis.^[28]^
2. Anticoagulation: Intravenous unfractionated heparin or low-molecular-weight heparin is administered to STEMI patients to prevent clot formation and extension during reperfusion therapy. It is often used in combination with antiplatelet therapy.^[1]^
3. Beta-blockers: Beta-blockers, such as metoprolol and carvedilol, are recommended in the acute phase of STEMI to reduce heart rate, blood pressure, and myocardial oxygen demand. They help in stabilizing the infarcted myocardium and preventing arrhythmias.^[27]^
4. Angiotensin-converting enzyme (ACE) Inhibitors and angiotensin receptor blockers (ARBs): These medications are prescribed in the early phase of STEMI to reduce ventricular remodeling, improve cardiac function, and prevent heart failure development.^[1]^
5. Statins: Early initiation of statin therapy is recommended in STEMI patients to reduce cholesterol levels and decrease the risk of recurrent cardiovascular events.^[29]^

#### 5.4.3. Cardiac rehabilitation.

Cardiac rehabilitation is an integral part of the management of STEMI patients. It involves a structured program that includes exercise training, education on heart-healthy lifestyle modifications, and psychosocial support. Cardiac rehabilitation has been shown to improve functional capacity, quality of life, and long-term outcomes in STEMI patients.^[30]^

In conclusion, the current treatment approaches for ST-Segment Elevation Myocardial Infarction focus on timely reperfusion therapy, using primary PCI as the preferred method whenever feasible. Adjunctive medical therapies reduce myocardial damage and improve patient outcomes, including dual antiplatelet therapy, anticoagulants, beta-blockers, ACE inhibitors or ARBs, and statins. Cardiac rehabilitation also plays a significant role in optimizing recovery and long-term management.

### 5.5. Guidelines for managing STEMI

Several reputable organizations have developed guidelines for managing STEMI. These guidelines provide evidence-based recommendations for the timely and effective diagnosis and treatment of STEMI patients.

#### 5.5.1. AHA and American College of Cardiology (ACC) guidelines for STEMI management.

The AHA/ACC guidelines provide comprehensive recommendations for managing STEMI, emphasizing the importance of timely reperfusion and adjunctive medical therapies. These guidelines highlight the role of primary PCI as the preferred reperfusion strategy when feasible, with a door-to-balloon time of 90 minutes or less for STEMI patients eligible for primary PCI.^[8]^ For patients who cannot undergo PCI within the recommended time frame, fibrinolytic therapy is an alternative if initiated within 30 minutes of hospital arrival.^[8]^ The guidelines also stress the importance of dual antiplatelet therapy, beta-blockers, ACE inhibitors or ARBs, and statins in managing STEMI patients.^[8]^

#### 5.5.2. ESC guidelines for the management of acute myocardial infarction in patients presenting with ST-segment elevation.

The ESC guidelines provide evidence-based recommendations for managing acute myocardial infarction, including STEMI. The guidelines emphasize the importance of rapid diagnosis and immediate reperfusion therapy, with a recommended door-to-balloon time of 90 minutes or less for primary PCI.^[1]^ The guidelines also guide the use of antiplatelet therapy, anticoagulation, beta-blockers, ACE inhibitors or ARBs, and statins in STEMI patients. Moreover, the ESC guidelines stress the significance of cardiac rehabilitation and secondary prevention measures in improving long-term outcomes and reducing recurrent cardiovascular events.^[1]^

The guidelines provided by the AHA, the ACC, and the ESC serve as valuable resources for healthcare professionals involved in the care of STEMI patients. These evidence-based recommendations underscore the critical importance of timely reperfusion therapy, optimal medical management, and cardiac rehabilitation in improving patient outcomes and reducing the burden of STEMI on global public health.

### 5.6. Prognosis

The prognosis of STEMI is influenced by several factors, including the extent of myocardial damage, the presence of complications, the effectiveness of reperfusion therapy, and the presence of comorbidities. Early and effective treatment and adherence to secondary prevention measures can significantly impact patient outcomes.

#### 5.6.1. Mortality.

STEMI is a severe and life-threatening condition; it can lead to substantial morbidity and mortality without prompt intervention. The mortality rate in STEMI patients varies depending on age, comorbidities, and the time to reperfusion therapy. Advances in reperfusion strategies and optimal medical management have improved survival rates. With timely reperfusion therapy, including primary PCI, mortality rates have decreased significantly.^[7]^

#### 5.6.2. Complications.

Complications following STEMI, such as heart failure, ventricular arrhythmias, cardiogenic shock, and mechanical complications (e.g., ventricular septal rupture or papillary muscle dysfunction), can significantly impact prognosis. The early identification and management of these complications are crucial in improving patient outcomes.^[31]^

#### 5.6.3. Left ventricular function.

The extent of myocardial damage in STEMI can lead to impaired left ventricular function. Left ventricular ejection fraction is an essential prognostic marker, with lower left ventricular ejection fraction values associated with a higher risk of adverse outcomes, including heart failure and mortality.^[32]^

#### 5.6.4. Recurrent cardiovascular events.

STEMI patients are at an increased risk of experiencing recurrent cardiovascular events, such as myocardial infarction, stroke, or unstable angina. Secondary prevention measures, including adherence to medication regimens and lifestyle modifications, are essential in reducing the risk of recurrent events.^[33]^

#### 5.6.5. Cardiac rehabilitation.

Participation in cardiac rehabilitation programs has improved functional capacity and quality of life and reduced mortality in STEMI patients. Regular exercise, education on risk factor modification, and psychosocial support contribute to better long-term outcomes.^[34]^

In conclusion, the prognosis of ST-Segment Elevation Myocardial Infarction is influenced by various factors, including myocardial damage, complications, and the effectiveness of reperfusion therapy. Early and effective treatment and adherence to secondary prevention measures can significantly impact patient outcomes and improve long-term prognosis.

## 7. Conclusion

STEMI represents a critical and life-threatening cardiovascular emergency that demands immediate attention and optimal management. The condition arises from the sudden occlusion of a coronary artery due to atherosclerotic plaque rupture and subsequent thrombus formation, leading to acute myocardial ischemia and necrosis. Prompt recognition and intervention are paramount to salvaging the ischemic myocardium and reducing morbidity and mortality.

Advances in reperfusion strategies, such as primary PCI, have revolutionized the treatment of STEMI patients, significantly improving survival rates and reducing long-term complications. The administration of adjunctive medical therapies, including dual antiplatelet therapy, anticoagulation, beta-blockers, ACE inhibitors or ARBs, and statins, further enhances outcomes and reduces the risk of recurrent events. The guidelines provided by renowned organizations, such as the AHA and the ESC, serve as invaluable resources, offering evidence-based recommendations to healthcare professionals involved in the care of STEMI patients.

Despite significant advancements in diagnosis and management, STEMI remains a considerable global health concern. Its epidemiology and risk factors vary across different regions and populations, with age, sex, lifestyle, and access to healthcare playing critical roles. Furthermore, the prognosis of STEMI patients is influenced by factors such as the extent of myocardial damage, complications, left ventricular function, and adherence to secondary prevention measures.

Early recognition and effective reperfusion therapy implementation, comprehensive medical management, and cardiac rehabilitation are essential to optimize patient outcomes. Furthermore, addressing risk factors, promoting healthy lifestyle choices, and ensuring timely access to medical care can significantly contribute to reducing the burden of STEMI on global health systems.

In pursuing better patient outcomes, research and innovation continue to drive improvements in the diagnosis, treatment, and management of STEMI. The evolving landscape of cardiovascular medicine offers hope for further advancements, with the ultimate goal of reducing the incidence, morbidity, and mortality associated with ST-Segment Elevation Myocardial Infarction.

## Author contributions

**Conceptualization:** Chukwuka Elendu, Dependable C. Amaechi, Tochi C. Elendu, Chisom E. Alakwe-Ojimba, Otite L. Akpovona, Yuliana Paola Oros Sucari, Kanishk Dang.

**Data curation:** Chukwuka Elendu, Dependable C. Amaechi, Tochi C. Elendu, Kanishk Dang.

**Formal analysis:** Chukwuka Elendu, Dependable C. Amaechi, Tochi C. Elendu.

**Funding acquisition:** Chukwuka Elendu.

**Investigation:** Chukwuka Elendu, Dependable C. Amaechi, Tochi C. Elendu, Eunice K. Omeludike.

**Methodology:** Chukwuka Elendu, Dependable C. Amaechi, Tochi C. Elendu, Chisom E. Alakwe-Ojimba, Yuliana Paola Oros Sucari.

**Project administration:** Chukwuka Elendu, Dependable C. Amaechi, Tochi C. Elendu, Babajide Obidigbo, Kanishk Dang.

**Resources:** Chukwuka Elendu, Dependable C. Amaechi, Tochi C. Elendu, Babajide Obidigbo.

**Software:** Chukwuka Elendu, Dependable C. Amaechi, Tochi C. Elendu, Sehajmeet Kaur Saggi, Chinedu P. Chinedu.

**Supervision:** Chukwuka Elendu, Dependable C. Amaechi, Tochi C. Elendu, Yuliana Paola Oros Sucari, Chinedu P. Chinedu.

**Validation:** Chukwuka Elendu, Dependable C. Amaechi, Tochi C. Elendu.

**Visualization:** Chukwuka Elendu, Dependable C. Amaechi, Tochi C. Elendu, Sehajmeet Kaur Saggi, Kanishk Dang.

**Writing – original draft:** Chukwuka Elendu, Dependable C. Amaechi, Tochi C. Elendu, Yuliana Paola Oros Sucari, Sehajmeet Kaur Saggi.

**Writing – review & editing:** Chukwuka Elendu, Dependable C. Amaechi, Tochi C. Elendu, Eunice K. Omeludike, Otite L. Akpovona, Yuliana Paola Oros Sucari, Sehajmeet Kaur Saggi, Kanishk Dang.
